# IDegLira improves time in range in a cohort of patients with type 2 diabetes: TiREX study

**DOI:** 10.1007/s00592-024-02361-7

**Published:** 2024-09-05

**Authors:** Maria Elena Malighetti, Laura Molteni, Emanuela Orsi, Roberta Serra, Alessia Gaglio, Federica Mazzoleni, Filomena Russo, Antonio Carlo Bossi

**Affiliations:** 1 Casa di Cura Ambrosiana - Piazza Monsignor Moneta 1, 20090 Cesano Boscone (MI), Italy; 2 Ospedale Sacra Famiglia Fatebenefratelli - via Fatebenefratelli 20, 22036 Erba (CO), Italy; 3 Fondazione IRCCS Ca’Granda Ospedale Maggiore Policlinico - via Francesco Sforza, 28–20122 Milano, Italy; 4 Fondazione Giuseppina Brunenghi - via Beccadello 6, 26012 Castelleone (CR), Italy; 5 Humanitas Gavazzeni - via Mauro Gavazzeni 21, 24125 Bergamo, Italy

**Keywords:** Type 2 diabetes, IDegLira, Glucometric indices, Time in range, Glucose variability

## Abstract

**Aims:**

To assess the effects of IDegLira on glucometric indices deriving from intermittently scanned Continuous Glucose Monitoring (isCGM) in patients with type 2 diabetes (T2D).

**Methods:**

Retrospective, observational, cohort, multi-center, “pre – post” study. All adults consecutively identified in the medical records who started treatment with IDegLira, and for whom an isCGM report before and after the initiation of IDegLira was available were included in the study. Time in range (TIR) represented the primary endpoint. Additional glucometric indices, insulin doses and body weight were also assessed.

**Results:**

Overall, 87 patients were included by 5 diabetes centers [mean age 70.2 ± 11.0 years, mean duration of T2D 15.5 ± 9.6 years; BMI 29.4 ± 5.4 kg/m^2^, baseline HbA1c 9.1 ± 2.1%, 33% insulin naïve, 20.7% treated with basal-oral therapy (BOT), and 46% treated with multiple daily injections of insulin (MDI)]. After an average of 1.7 weeks from IDegLira initiation, TIR significantly increased from 56.8 ± 23.5% to 81.3 ± 13.5% (*p* < 0.0001), TAR decreased from 42.3 ± 24.2% to 17.1 ± 13.6% (*p* < 0.0001), while TBR remained steadily low (from 1.3 ± 2.3% to 1.4 ± 2.6%; *p* = 0.62). Estimated HbA1c decreased from 9.1 ± 2.1% to 6.7 ± 0.6% (*p* < 0.0001) and percentage of patients with a blood glucose coefficient of variation ≥ 36% dropped from 33.2 to 13.8% (*p* = 0.0005). In patients on MDI, the reduction in the total insulin dose was substantial (from 55.8 ± 31.2 IU to 27.2 ± 12.3 U).

**Conclusions:**

In T2D patients with poor metabolic control, either insulin naïve or treated with BOT or MDI, the introduction of IDegLira produces a significant increase in the time spent in good metabolic control and a marked reduction in glycemic fluctuations.

**Supplementary Information:**

The online version contains supplementary material available at 10.1007/s00592-024-02361-7.

## Introduction

Despite the availability of new classes of glucose-lowering drugs, insulin therapy still represents a cornerstone of the treatment for type 2 diabetes mellitus (T2D), and many patients eventually require and benefit from insulin therapy.

Typically, patients with newly diagnosed T2D are encouraged by their doctors to follow a proper diet and improve their lifestyle. However, T2D is a progressive disease which almost always requires intensification of treatment. The first pharmacological step usually involves the addition of oral glucose lowering drugs, often followed by glucagon-like peptide-1 receptor agonists (GLP-1 RAs) (injectable or as oral formulation) in people not attaining their glycemic goals.

The addition of basal insulin to oral treatment is a well-established approach [1]. Patients may be reluctant to intensify treatment, particularly when insulin is involved, due to concerns about possible hypoglycemic episodes, body weight gain and/or the complexity of such regimens. The same type of problems is also found in patients in a more advanced phase of the disease, when insulin has already been included in the therapeutic plan, aggravated by the fact that complex administration schedules require several daily injections, considered by 23.1% of patients with T2D as a psychophysical obstacle to daily life [2]. For this reason, different types of combinations between oral agents and basal insulin analogue (Basal-oral therapy, BOT) are often utilized in clinical practice. However, BOT, and even more so multiple daily injections (MDI), are burdened by the risk of hypoglycemia [3], the increase in body weight [4] and the variability of glycemic control [5].

Patients are scared of hypoglycemia, which represents the main concern also for diabetologists: it has been estimated that 57.9% of patients with T2D reduce their insulin dose after the onset of severe hypoglycemia [6] and 79.1% of physicians indicate that the risk of hypoglycemia prevents them from initiating more aggressive treatment [2]. It is therefore not surprising that in some studies approximately half of T2D patients treated with basal insulin do not reach the glycated hemoglobin (HbA1c) target [5, 7–9]. The anabolic effect of insulin and the consequent weight increase, in addition to penalizing the achievement of the HbA1c target [4, 5, 7, 8], induce an increase in the risk of cardiovascular diseases, already accentuated by the condition of dysglycemia [10]. Finally, evidence has emerged in support of glycaemic variability as an independent risk factor for diabetes microvascular and macrovascular complications, total mortality, and death due to cardiovascular disease in both type 1 and type 2 diabetes [11].

Insulin degludec/liraglutide (IDegLira) (Xultophy^®^ Novo Nordisk A/S, Bagsværd, Denmark), is a fixed-ratio combination of Insulin Degludec and Liraglutide (a GLP1 hormone receptor agonist) administered once a day. The pharmacokinetic properties of the two individual components, insulin Degludec and Liraglutide, are preserved in the fixed-ratio combination product IDegLira [12]. The complementary action of the two drugs seems to be indicated as the start of insulin therapy, since the efficacy of insulin is associated with the safety profile of the GLP-1 analogue. Particularly, in randomized clinical trials (RCTs) comparing IDegLira to Insulin Glargine, a reduction in the risk of hypoglycaemia was documented, with little or no weight gain and longer durability of treatment effect [13, 14].

In patients already using insulin, in phase III studies, IDegLira administered once a day resulted in significantly lower HbA1c levels and a loss of body weight compared to weight gain with Insulin Glargine U100 [13]. Therefore, when treatment with BOT has an insufficient therapeutic effect, IDegLira once a day can improve glycemic control with a reduced risk of hypoglycemia, with no weight gain.

Finally, in patients on multi-injection insulin therapy, a RCT demonstrated that treatment with IDegLira was responsible for a reduction in HbA1c comparable to MDI, with statistically significant lower rates of hypoglycemia; body weight decreased with IDegLira and increased with MDI [15].

Continuous glucose monitoring (CGM) devices are increasingly used to collect data to complement observations of the effects of therapeutic interventions in diabetes [16].

However, it still remains a gap in knowledge on the effects of this drug, since there are not so many data from RCTs or real-world evidence on the effect of IDegLira on CGM-derived glucometric indices and few information on glycemic variability. Therefore, the purpose of Time in Range Evaluation in Xultophy therapies (TiREX) study was to assess the effects of IDegLira on the glucometric indices deriving from intermittently scanned Continuous Glucose Monitoring (isCGM), with particular focus on blood glucose fluctuations.

## Methods

### Study objectives

The aim of TiREX study was to evaluate the average changes in glycemic control in adults with T2D, measured as Time-in-Range, Time-above-Range and Time-below-Range, and changes in glucose variability, measured as coefficient of variation (CV) before and after the initiation of IDegLira.

The modification of therapy in these patients was based on clinical judgement, and the choice of the new drug was driven by multiple reasons, which can be recognized mainly in the intent:

- To improve the metabolic control, reaching the HbA1c targets recommended by national and international guidelines.
- To reduce the risk of hypoglycemia typical of intensive insulin therapies.
- To counteract the weight gain associated with the anabolic effect of insulin.
- To limit the problem of patients’ poor adherence to multi-injection therapies.
- To facilitate the correct titration of drugs by the patients, typically hindered by the complex therapeutic schemes of chronic therapies.

### Study design

This was a retrospective, observational, cohort, multi-center, “pre – post” study. Data were extracted from the medical records of the diabetes centers participating in the study.

The decision to start treatment with IDegLira was made according to the judgment of the clinicians and was independent of the subsequent decision to include the patients in the study.

No diagnostic or monitoring procedures outside of normal clinical practice were applied to the patients.

Given the retrospective design, all adults with T2D consecutively identified in the medical records who started treatment with IDegLira within the defined time interval (from 01/01/2023 until 06/30/2023), and for whom an isCGM report was available were included in the study. A previous treatment with GLP1-RA represented the only exclusion criteria.

### Study endpoints

The primary endpoint of the study was to assess the effectiveness of IDegLira, when administered according to clinical practice in adults with T2D and unsatisfactory metabolic control, on the Time-in-Range (TIR) glycemic parameter.

The pre-post variation of the following secondary endpoints was also considered:

- Time spent in hypoglycemia (time below range, TBR < 70 mg/dl).
- Time spent in hyperglycemia (time above range, TAR > 180 mg/dl).
- Average blood glucose and estimated HbA1c.
- Glycemic variability (coefficient of variation).
- Insulin doses administered (IU/day).
- Body weight.

### Study procedures

Glucometric indices were detected using an intermittently scanned Continuous Glucose Monitoring (isCGM) sensor (Freestyle Libre^®^ Abbott Diabetes Care, Abbott Park, IL, USA). In particular, the observations refer to two periods of glycemic data collection: the first period before initiation of IDegLira, and the second following the initiation of therapy with IDegLira. Each period lasted a minimum of three consecutive weeks. The two observation periods could be separated by a few weeks during which the dosage of IDegLira was titrated and optimized for each patient.

Patients were instructed in the use of the prescribed devices (glycemic sensor and insulin injection pen) by the medical-nursing staff of the Diabetes Center.

The study was approved by the Ethics Committees of the participating centers. Patients signed an informed consent for authorizing the retrospective use of their data.

### Statistical methods

A paired design was used to test whether the paired difference in distributions of TIR (δ) was different from 0 (H0: δ = 0 versus H1: δ ≠ 0). The comparison was made using a two-sided, paired-difference Wilcoxon Signed-Rank test, with a Type I error rate (α) of 0.05. The underlying data distribution of paired differences was assumed to be normal. The underlying standard deviation of the paired difference distribution was assumed to be 30. To detect a paired mean difference of TIR of 10% with 80% power, the number of needed pairs was 77.

Descriptive data were reported as mean and standard deviation in case of continuous variables, or proportions in case of categorical variables. Pre-post comparisons were performed using the Wilcoxon Signed-Rank test (continuous variables) or the McNemar χ^2^ test (categorical variables).

## Results

Overall, 87 patients were included by 5 diabetes centers. Patient characteristics are reported in Table 1. Participants had a mean age of 70.2 ± 11.0 years and a mean duration of T2D of 15.5 ± 9.6 years; the mean BMI was 29.4 ± 5.4 kg/m^2^. Baseline HbA1c levels (9.1 ± 2.1%) and fasting blood glucose levels (191.0 ± 75.0 mg/dl) testified to the poor metabolic control of these patients. One-third of participants were insulin naïve (33.3%), 20.7% were treated with BOT, and 46% were treated with MDI schemes.

**Table 1:** Patient characteristics, overall and by therapeutic scheme

Characteristics	Overall	Insulin naïve	BOT	MDI
N.	87	29	18	40
Males (%)	59.8	55.2	55.6	65.0
Age (years)	70.2 ± 11.0	69.3 ± 13.4	68.6 ± 7.3	71.6 ± 10.5
Duration of T2D (years)	15.5 ± 9.6	12.6 ± 8.5	11.3 ± 6.4	19.5 ± 10.2
HbA1c (%)	9.1 ± 2.1	10.1 ± 2.1	8.9 ± 1.6	8.4 ± 2.0
Fasting blood glucose (mg/dl)	191.0 ± 75.0	222.9 ± 68.9	181.8 ± 99.1	167.9 ± 60.3
Body weight (Kg)	80.5 ± 15.9	77.3 ± 16.4	79.8 ± 15.2	83.2 ± 15.7
BMI (Kg/m^2^)	29.4 ± 5.4	28.0 ± 4.9	28.7 ± 5.7	30.8 ± 5.3
eGFR (ml/min/1.73 m^2^)	56.7 ± 22.1	62.2 ± 20.7	62.5 ± 19.1	50.2 ± 22.9

Compared to insulin naïve and BOT patients, MDI patients were more frequently men, were older, with longer diabetes duration and higher BMI. On the other hand, insulin naïve patients showed poorer metabolic control.

The most frequently prescribed oral agents were metformin (66.7%) and SGLT2i (46.0%). In BOT patients, the mean dose of basal insulin was 18.8 ± 9.8 IU/day; in MDI patients, the mean dose of basal insulin was 27.3 ± 14.5 IU/day and the mean dose of rapid acting insulin was 28.5 ± 19.5 IU/day.

As for isCGM derived glycemic indices, before the initiation of treatment with IDegLira, mean TIR, TAR and TBR were 56.8 ± 23.5%, 42.3 ± 24.2%, and 1.3 ± 2.3%, respectively. Mean blood glucose levels were 175.6 ± 36.5 mg/dl, with a coefficient of variation of 32.0 ± 7.2%.

IDegLira was initially prescribed at a mean dose of 16.6 ± 7.0 U (0.2 U/kg).

The two observation periods were separated by a median of 1 week (IQR 1–2) during which the dosage of IDegLira was titrated and optimized for each patient. This short titration period is motivated by the continuous support provided to patients by healthcare staff to achieve personalized baseline glycemic goals. Compared to treatment before the initiation of IDegLira, no significant changes in the use of oral agents were documented (Table 2). In BOT, the basal insulin dose used only slightly increased after the switch to IDegLira (from 18.8 ± 9.8 IU of basal insulin to 20.2 ± 9.3 U of IDegLira), while the reduction in the total insulin dose was substantial, thanks to the suspension of rapid acting insulin, in patients on MDI (from 55.8 ± 31.2 IU to 27.2 ± 12.3 U).

**Table 2:** Treatment characteristics at baseline (T0) and after initiation of IDegLira (T1), by therapeutic scheme

Characteristics	T0	T1	*p*
IDegLira (U)			
Overall	16.6 ± 7.0	23.0 ± 10.9	< 0.0001
Insulin naïve	17.8 ± 7.8	19.0 ± 7.3	0.06
BOT	15.9 ± 4.9	20.2 ± 9.3	0.02
MDI	16.0 ± 7.3	27.2 ± 12.3	< 0.0001
IDegLira (U/Kg)			
Overall	0.21 ± 0.08	0.30 ± 0.13	< 0.0001
Insulin naïve	0.23 ± 0.09	0.33 ± 0.09	0.03
BOT	0.20 ± 0.06	0.26 ± 0.10	0.01
MDI	0.20 ± 0.08	0.34 ± 0.15	< 0.0001
Basal insulin dose (IU)			
Insulin naïve	-	-	-
BOT	18.8 ± 9.8	0	-
MDI	27.3 ± 14.5	0	-
Rapid acting insulin dose (IU)			
Insulin naïve	-	-	-
BOT	-	-	-
MDI	28.5 ± 19.5	0	-
Metformin (%)			
Overall	66.7	63.2	0.26
Insulin naïve	86.2	75.9	0.19
BOT	88.9	88.9	1.00
MDI	42.5	42.5	1.00
Sulphonylureas/glinides (%)			
Overall	3.4	1.1	0.34
Insulin naïve	6.9	0	-
BOT	5.6	0	-
MDI	0	2.5	-
Pioglitazone (%)			
Overall	2.3	4.6	0.17
Insulin naïve	3.4	6.9	0.34
BOT	5.6	5.6	1.00
MDI	0	2.5	-
DPP4i (%)			
Overall	3.4	0	-
Insulin naïve	6.9	0	-
BOT	5.6	0	-
MDI	0	0	-
SGLT2i (%)			
Overall	46.0	44.8	0.71
Insulin naïve	37.9	41.4	0.33
BOT	44.4	38.9	0.58
MDI	52.5	50.0	0.57

In all subgroups (insulin naïve, BOT, MDI) the dose of IDegLira was up-titrated to 0.3 U/kg. As for isCGM derived glycemic indices, TIR significantly increased from 56.8 ± 23.5% to 81.3 ± 13.5% (*p* < 0.0001), with a parallel decrease of TAR (from 42.3 ± 24.2% to 17.1 ± 13.6%; *p* < 0.0001), while TBR remained steadily low (from 1.3 ± 2.3% to 1.4 ± 2.6%; *p* = 0.62). Estimated HbA1c decreased from 9.1 ± 2.1% to 6.7 ± 0.6% (*p* < 0.0001). A significant reduction in fasting blood glucose, mean blood glucose and blood glucose coefficient of variation (CV) was also documented (Table 3). In particular, the proportion of patients with a CV ≥ 36% dropped from 33.2 to 13.8% (*p* = 0.0005). The reduction in CV was particularly evident in the MDI group (from 25.0 to 7.5%; *p* = 0.01). Finally, body weight significantly decreased from 80.5 ± 15.9 Kg to 79.3 ± 16.1 Kg (*p* = 0.02), with a parallel reduction of BMI.

**Table 3:** isCGM derived glucometric measures and anthropometric data at baseline (T0) and after initiation of IDegLira (T1), overall and by therapeutic scheme

Characteristics	T0	T1	*p*
Time in range (%)			
Overall	56.8 ± 23.5	81.3 ± 13.5	< 0.0001
Insulin naïve	47.1 ± 24.4	82.2 ± 13.3	< 0.0001
BOT	61.4 ± 23.2	78.8 ± 16.3	0.0001
MDI	61.8 ± 21.4	81.7 ± 12.5	< 0.0001
Time above range (%)			
Overall	42.3 ± 24.2	17.1 ± 13.6	< 0.0001
Insulin naïve	52.3 ± 24.8	17.1 ± 13.3	< 0.0001
BOT	39.3 ± 23.7	19.8 ± 16.1	< 0.0001
MDI	36.5 ± 22.1	16.0 ± 12.7	< 0.0001
Time below range (%)			
Overall	1.3 ± 2.3	1.4 ± 2.6	0.62
Insulin naïve	0.6 ± 1.1	0.9 ± 2.0	0.44
BOT	1.2 ± 3.3	0.9 ± 1.9	0.60
MDI	1.9 ± 2.3	2.0 ± 3.1	0.73
HbA1c (%)*			
Overall	9.1 ± 2.1	6.7 ± 0.6	< 0.0001
Insulin naïve	10.1 ± 2.1	6.7 ± 0.7	< 0.0001
BOT	8.9 ± 1.6	6.7 ± 0.8	0.0001
MDI	8.4 ± 2.0	6.7 ± 0.6	< 0.0001
Fasting blood glucose (mg/dl)			
Overall	191.0 ± 75.0	119.1 ± 23.0	< 0.0001
Insulin naïve	222.9 ± 68.9	121.9 ± 23.1	< 0.0001
BOT	181.8 ± 99.1	116.3 ± 27.0	0.015
MDI	167.9 ± 60.3	118.2 ± 21.1	0.0001
Mean blood glucose levels (mg/dl)			
Overall	175.6 ± 36.5	140.6 ± 27.1	< 0.0001
Insulin naïve	184.0 ± 28.8	143.6 ± 35.2	< 0.0001
BOT	168.1 ± 34.3	140.3 ± 24.9	< 0.0001
MDI	173.0 ± 41.7	138.6 ± 21.2	< 0.0001
Coefficient of variation (%)			
Overall	32.0 ± 7.2	28.2 ± 6.7	< 0.0001
Insulin naïve	30.3 ± 9.0	28.9 ± 7.0	0.25
BOT	31.9 ± 6.6	28.0 ± 6.4	0.001
MDI	33.3 ± 5.8	27.7 ± 6.7	< 0.0001
Coefficient of variation ≥ 36%			
Overall	33.2	13.8	0.0005
Insulin naïve	35.7	20.7	0.1
BOT	33.3	16.7	0.09
MDI	25.0	7.5	0.01
Body weight (Kg)			
Overall	80.5 ± 15.9	79.3 ± 16.1	0.02
Insulin naïve	77.3 ± 16.4	76.0 ± 15.7	0.002
BOT	79.8 ± 15.2	78.2 ± 14.7	0.007
MDI	83.2 ± 15.7	82.2 ± 16.7	0.35
BMI (Kg/m^2^)			
Overall	29.4 ± 5.4	29.0 ± 5.3	0.006
Insulin naïve	28.0 ± 4.9	27.6 ± 4.6	0.002
BOT	28.7 ± 5.7	28.1 ± 5.5	0.006
MDI	30.8 ± 5.3	30.4 ± 5.3	0.22

*At T1 HbA1c levels were estimated by isCGM

## Discussion

### Major findings

The use of IDegLira led to a significant increase in TIR values, with a marked reduction in TAR, without an increase in the risk of hypoglycemic events, as supported by the low, stable TBR. The 25% increase in TIR translates into 6 more hours spent with glycemic values in range, with a parallel reduction in the time spent with elevated glycemic values. The significant increase in TIR occurred in all treatment groups, either insulin naïve, BOT, or MDI. The glycated hemoglobin value estimated by the sensor in the two weeks following the titration was significantly improved, confirming an improvement in blood glucose control. Significant reductions in average and fasting blood glucose levels were also documented.

The use of IDegLira also determined a reduction in blood glucose variability. Available evidence shows that a CV value < 36% represents low glucose variability and a relatively stable glucose profile, whereas a CV value ≥ 36% indicates an unstable glucose profile [17, 18]. In our study, a marked reduction in the proportion of patients with elevated CV was documented after the initiation of IDegLira, particularly among patients previously treated with MDI. Despite the short period of observation, a statistically significant reduction in body weight and BMI was also documented overall, in insulin naïve patients, and in those treated with BOT.

### Comparison with existing evidence

Evidence on the impact of treatment with IDegLira on CGM-derived glucometric indices is scant. A post hoc analysis of the DUAL V and DUAL VIII trials compared derived-TIR, calculated from self-monitored blood glucose, with IDegLira versus insulin glargine 100 units/mL (glargine U100) in people with T2D [19]. Nine-point SMBG profiles were taken over one day at baseline and end of trial (EOT: 26 weeks [DUAL V] and 104 weeks [DUAL VIII]). Changes from baseline to EOT in TIR were significantly greater with IDegLira versus glargine U100 both in DUAL V (4.18%, *P* = 0.027) and DUAL VIII (5.17%, *P* = 0.001) [13, 14].

An observational, multicenter study evaluated the effectiveness and safety of switching from basal-bolus to IDegLira in 234 patients with T2D who had preserved insulin secretion but inadequate glucose control [20]. In a subgroup of 55 patients who underwent CGM, a significant increase in TIR (57.9% vs. 69.0%; *p* < 0.01) and a decrease in TAR (40.1% vs. 28.8%; *p* < 0.01) were documented, while TBR, hypoglycemia (number of episodes per patient and proportion of patients), and glucose variability did not change significantly. Furthermore, recent studies, including those by Di Loreto et al. (2024) [21] and Fadini et al. (2024) [22], have already provided comprehensive data on the effectiveness and persistence of IDegLira treatment in real-world settings over extended periods. These studies, along with the work of Kawaguchi et al. (2024) [23] and Oya et al. (2024) [24], not only incorporate control or comparative treatment groups but also offer a more thorough investigation into the long-term benefits and comparative effectiveness of IDegLira versus other therapeutic combinations.

### Implications for clinical practice

Our study shows that the initiation of treatment with IDegLira improves two key risk factors for diabetes complications, namely poor metabolic control and glucose variability. These benefits are added to the well-recognized cardio-renal protective effects of GLP1-RAs [25]. The reduction in glucose variability deserves particular consideration, in the light of the large body of evidence linking glucose fluctuations to the risk of diabetes complications and mortality [11]. In addition, the improvements in endothelial dysfunction, inflammation, oxidative stress and the immunological properties of GLP1-RAs related to their mechanism of action could contribute to the positive effects of this drug class [26–29].

The initiation of IDegLira also presents additional advantages. In insulin naïve patients with very poor metabolic control, it allows to initiate an injection therapy with minimal risk of hypoglycemia and weight gain, thus overcoming the reluctance to therapy intensification of patients and diabetologists, and reducing clinical inertia [30]. In patients treated with BOT, therapy intensification with IDegLira would allow the avoidance of complex MDI schemes, associated with increased risk of hypoglycemia and weigh gain and patient discomfort. Similarly, in patients treated with MDI, the initiation of IDegLira would permit the suspension of rapid acting insulin, thus leading to therapy simplification and substantial reduction in total insulin doses, with its associated benefits. Also, from the patient point of view, IDegLira may offer appeal based on the need for fewer injections, less required self-monitoring compared to basal-bolus insulin regimens, and the potential to limit excess use of basal insulin.

### Strengths and limitations

Our study has strengths and limitations. Among the strengths, this study adds important insights regarding the impact of IDegLira on CGM-derived glucometric indices and confirms the favorable evidence on its effect new evidence on its effect on glucose variation. The major limitations are represented by the small number of participants and the lack of long-term data documenting the persistence of the positive effects described in our study.

## Conclusions

In conclusion, in T2D patients with poor metabolic control, either insulin naïve or treated with BOT or MDI schemes, the introduction of IDegLira produces not only a significant improvement of metabolic control, but also a marked reduction in glycemic fluctuations, which are demonstrated to have a major impact on the occurrence of diabetes-related micro- and macroangiopathic comorbidities.

## Electronic supplementary material

Below is the link to the electronic supplementary material.

Supplementary Material 1

## Data Availability

The datasets generated during and/or analyzed during the current study are available from the corresponding author on reasonable request.

## Abbreviations

BMI: Body Mass Index
BOT: Basal-Oral Therapy
CGM: Continuous Glucose Monitoring
CV: Coefficient Of Variation
GLP-1: RAs-Glucagon-Like Peptide-1 Receptor Agonists
HbA1c: Glycated Hemoglobin
IDegLira: Insulin Degludec/Liraglutide
isCGM: Intermittently Scanned CGM
MDI: Multiple Daily Injections
RCT: Randomized Clinical Trial
T2D: Type 2 Diabetes Mellitus
TAR: Time Above Range
TBR: Time Below Range
TIR: Time-In-Range
T2D: Type 2 Diabetes Mellitus

## Declarations

### Medical writing and/or editorial assistance

Editorial assistance provided by CORESEARCH SRL (Maria Chiara Rossi, Giusi Graziano) and the Open Access fee was funded through a Novo Nordisk S.p.A. unconditional grant. The authors of the publication are fully responsible for the contents and conclusions. Novo Nordisk S.p.A. did not influence and has not been involved in the data interpretation presented in the manuscript.
